# Efficient Removal of Polyvalent Metal Ions (Eu(III) and Th(IV)) from Aqueous Solutions by Polyurea-Crosslinked Alginate Aerogels

**DOI:** 10.3390/gels8080478

**Published:** 2022-07-29

**Authors:** Efthalia Georgiou, Ioannis Pashalidis, Grigorios Raptopoulos, Patrina Paraskevopoulou

**Affiliations:** 1 Laboratory of Radioanalytical and Environmental Chemistry, Department of Chemistry, University of Cyprus, P.O. Box 20537, Cy-1678 Nicosia, Cyprus; georgiou.efthalia@ucy.ac.cy; 2 Inorganic Chemistry Laboratory, Department of Chemistry, National and Kapodistrian University of Athens, Panepistimiopolis Zografou, 15771 Athens, Greece; grigorisrap@chem.uoa.gr

**Keywords:** alginate aerogels, polymer-crosslinked aerogels, polyurea-crosslinked alginate aerogels, Th(IV) sorption, Eu(III) sorption, thermodynamic, environmental remediation, water decontamination

## Abstract

The removal of polyvalent metal ions Eu(III) and Th(IV) from aqueous solutions using polyurea-crosslinked calcium alginate (X-alginate) aerogels has been investigated by batch-type experiments under ambient conditions and pH 3. The material presents relatively high sorption capacity for Eu(III) (550 g kg^−1^) and Th(IV) (211 g kg^−1^). The lower sorption capacity for Th(IV) compared to Eu(III) is attributed to the net charge of the dominant species in solution under the given experimental conditions, which is Eu^3+^ for Eu(III), and Th(OH)_2_^2+^ and Th(OH)_3_^+^ for Th(IV). Generally, the sorption is an endothermic and entropy-driven process, and it follows the Langmuir isotherm model. According to the FTIR spectra, sorption occurs via formation of inner-sphere complexes between the surface functional groups and the *f*-metal cationic species. The presence of europium and thorium in the adsorbent material was confirmed and quantified with EDS analysis. To the best of our knowledge, this is the first report of an aerogel material used as an adsorbent for Eu(III). Compared to other materials used for the sorption of the specific ions, which are mostly carbon-based, X-alginate aerogels show by far the highest sorption capacity. Regarding Th(IV) uptake, X-alginate aerogels show the highest capacity per volume (27.9 g L^−1^) among the aerogels reported in the literature. Both Eu(III) and Th(IV) could be recovered from the beads by 65% and 70%, respectively. Furthermore, Th(VI) could also be quantitatively removed from wastewater, while Eu(III) could be removed by 20%. The above, along with their stability in aqueous environments, make X-alginate aerogels attractive candidates for water treatment and metal recovery applications.

## 1. Introduction

Europium is a member of the lanthanide series and one of the rarest members of the rare earth elements. Similar to other lanthanide elements, europium in aqueous solutions exists basically in the trivalent oxidation state and, under ambient conditions, hydrolysis and carbonate complexation determine its chemical behavior. Although a heavy metal, europium is relatively non-toxic, because it has no significant role in biological processes. Europium compounds have found industrial applications because of their optical/luminescent properties. In addition, Eu(III) is used as an analogue for trivalent lanthanides and actinides, such as Am(III) and Cm(III), because it presents similar aquatic chemistry to its radioactive counterparts, is non-(radio)toxic and possesses useful fluorescent properties, which make spectroscopic studies on its chemical behavior and speciation possible [1].

Efficient collection of lanthanides (including europium) from process waters and industrial wastewaters prior to their discharge into environmental receivers is nowadays mandatory in order to protect the environment and secure the future availability of lanthanide resources. Lanthanides play a main role in the fabrication of different industrial products (e.g., automotive catalysts, magnets, optical devices, ceramics, etc.), as well as in green and sustainable energy production [2,3].

Thorium belongs to the actinide series of elements. Monazite contains relatively high amounts of thorium together with other rare earth elements, and it is extracted from the ore by liquid−liquid extraction using tributyl phosphate [4]. The naturally occurring thorium isotope (Th-232) is a weakly radioactive nuclide, and not fissile, but it can be transmuted via neutron absorption into the fissile uranium isotope U-233. Over the next decades thorium could play a major role as a nuclear fuel in liquid fluoride thorium reactors (LFTR), which, compared to uranium-fueled light water reactors, are significantly more energy effective, and produce far less nuclear waste [5]; and they are also characterized of higher safety, which is associated with considerably lower chances of getting into a nuclear accident [6]. In addition, thorium is more abundant than uranium, which results to lower energy production prices [6].

In the past, thorium has been widely used in ceramic glazes, lantern mantles, and welding rods. In addition, thorium in the form of colloidal thorium dioxide was used until the 1950s as a contrast agent in medical radiology. Studies of people and patients who were exposed to thorium have shown increased risk of liver tumors, pancreatic, lung, and bone cancer. The latter is mainly because thorium is stored in bones [7]. It is expected that increased levels of thorium in the biosphere could have negative effects on the environment and human health [8]. Therefore, removal of thorium from industrial process waters and wastewaters prior to their discharge into environmental receivers is of particular importance regarding the protection of the associated ecosystems. In addition, possible recovery of thorium from industrial wastewaters is of particular interest because of its use in nuclear fuel production.

Metal removal technologies based on adsorption have attracted a significant interest because of their ease of operation, possible reuse of absorbents, and efficient recovery of precious elements [9]. Aerogel materials as adsorbents are very attractive because of their diverse chemical composition, high porosity, and a wide range of pore dimensions, including micro-, meso-, and macropores, resulting in effective and low-cost processes, and often also in selectivity towards the desirable adsorbates [10,11].

One example of aerogel materials studied as adsorbents are the polyurea-crosslinked alginate (X-alginate) aerogels, which are a new class of biopolymer-based aerogels with high mechanical strength [12,13,14]. These aerogels were prepared according to the polymer-cross-linked (X-aerogel) technology [15,16,17,18,19,20], by the reaction of aliphatic or aromatic triisocyanates with pre-formed M-alginate wet-gels (M refers to divalent or trivalent metal cation), which results in the formation of a nano-thin layer of polyurea over the M-alginate skeleton, leaving the primary M-alginate structure practically undisturbed. That polyurea layer renders the materials hydrophobic, in contrast to the corresponding M-alginate aerogels that are extremely hydrophilic. The nature of polyurea (aliphatic or aromatic) is also significant, as the aliphatic polyurea layer is more compact compared to the aromatic polyurea layer, which is a more randomly oriented polymer structure, leaving more exposed the coordination sites [21].

**Figure 1:**
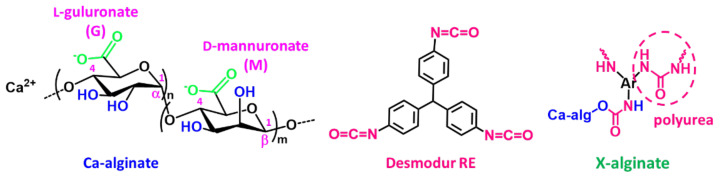
The structures of Ca-alginate, triphenylmethane-4,4′,4″-triisocyanate (TIPM; Desmodur RE) and the corresponding X-alginate aerogels. Ca-alginate is a block copolymer of β-(1→4)-linked D-mannuronate (M) and α-(1→4)-linked L-guluronate (G).

Based on the above, X-alginate aerogels (Figure 1) derived from pre-formed Ca-alginate (Figure 1) gels and an aromatic triisocyanate (Desmodur RE; Figure 1) have been found to be excellent candidate materials for environmental remediation. These materials combine hydrophobicity, high mechanical strength, and high stability (no swelling, shrinking, or disintegration) in all aqueous environments, including seawater. Therefore they have been used for decontamination of seawater from Pb(II), organic solvents, and oil [22], and also for U(VI) uptake from various water environments [23]. In the case of U(VI), the adsorption process was especially fast, and X-alginate aerogels showed an extremely high adsorption capacity uptake—twice the mass of the material, which compared to that of other aerogel materials is one of the highest sorption capacities per weight and the highest per volume. In addition to this, uranium could be recovered almost quantitatively.

In this study, X-alginate aerogels described above have been studied in terms of their ability to sorb Eu(III) and Th(IV) from aqueous solutions. In addition to batch-type experiments, which were carried out to investigate the effect of various parameters on the sorption efficiency of the aerogels towards Eu(III) and Th(IV), FTIR and EDS data were used to confirm the post-adsorption presence of Eu(III) and Th(IV) on the adsorbent and to evaluate the sorption mechanism.

## 2. Results and Discussion

### 2.1. Eu(III) Sorption

Τhe adsorption capacity of the X-alginate aerogels for Eu(III) has been studied with batch-type experiments, under ambient conditions and at pH 3.0. The experiments were carried out at pH 3.0 to avoid the formation of hydrolysis products and enable the use of increased metal ion concentrations without considering complex polynucleation and surface precipitation reactions. The dominant species in solution under the given experimental conditions is Eu^3+^. The initial Eu(III) concentration varied between 10^−^^5^ and 0.1 mol L^−1^, and the isothermal data were fitted with the Langmuir model (Equation (1)), where *q*_e_ is the Eu(III) uptake (in mol kg^−1^) at equilibrium, *C*_e_ is the Eu(III) concentration in the solution at equilibrium (in mol L^−1^), *q*_max_ is the adsorption capacity (in mol kg^−1^), and *K*_L_ the *Langmuir* equilibrium constant (in L mol^−1^). Equation (1) is as follows:

(1) $$q_{e}=\frac{q_{\max}\;K_{L}\;C_{e}}{1+K_{L}\;C_{e}}$$

**Figure 2:**
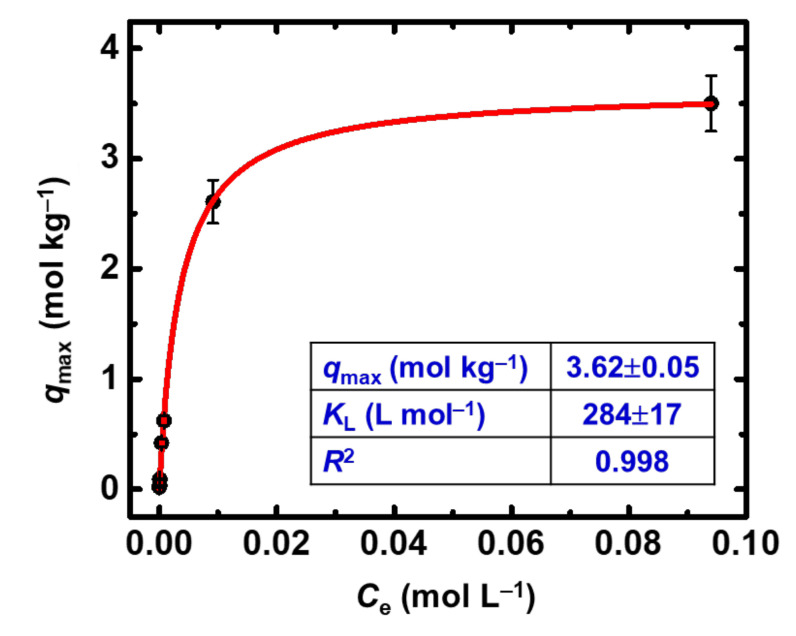
Experimental data and Langmuir sorption isotherm of Eu(III) on X-alginate aerogels at 283 K and varying initial Eu(III) concentrations (10^−5^–0.1 mol L^−1^). Experimental conditions are as follows: contact time 24 h, pH 3.0, adsorbent dosage 0.4 g L^−1^, agitation rate 125 rpm.

The isothermal data are shown in Figure 2; these data are best fitted with the Langmuir isotherm model, which assumes that specific sites are available for Eu(III) coordination. The plateau of this curve is associated with saturation of these sites, which occurs at increased Eu(III) concentrations. Evaluation of the experimental data by the Langmuir isotherm model resulted in an adsorption capacity (*q*_max_) of 3.62 mol Eu(III) per kg of X-alginate aerogel (550 g kg^−1^), which is a supreme value when considering corresponding literature values (90 g kg^−1^ < *q*_max_ < 360 g kg^−1^) for carbon-based adsorbents, particularly under acidic conditions (pH 3) [24,25].

**Figure 3:**
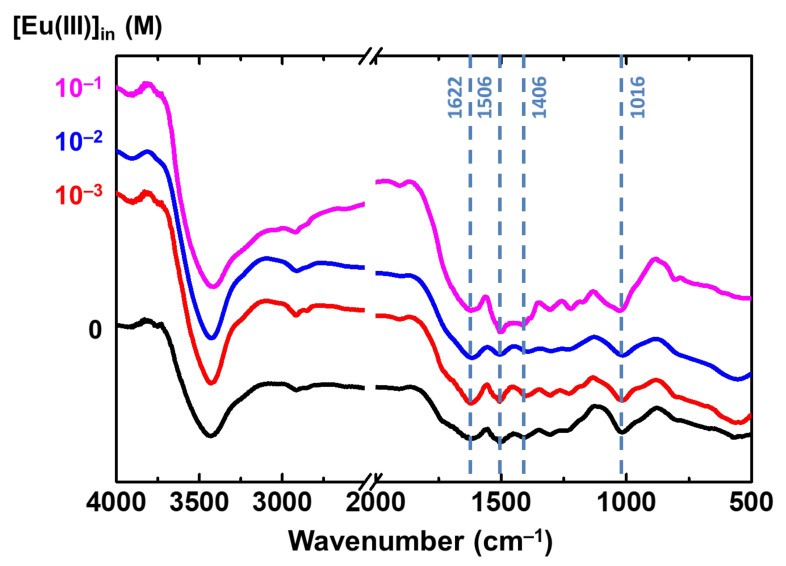
FTIR spectra of X-alginate aerogel beads (black line) and dried X-alginate beads after sorption of different amounts of Eu(III) (colored lines) from solutions with different initial Eu(III) concentrations (10^−3^–0.1 mol L^−1^, as indicated).

The FTIR spectra in Figure 3, which correspond to X-alginate aerogel beads (black line) and dried X-alginate beads after sorption of different amounts of Eu(III) (colored lines), clearly show that the shape and the relative intensity of the bands at 1622 and 1406 cm^−1^ (attributed to –COO^−^ coordinated to Ca(II)) [26], 1506 cm^−1^ (attributed to –NH groups [14]) and 1016 cm^−1^ (attributed to –C–O–C– groups of the sugar ring [14]) change upon increasing Eu(III) adsorption. This is an indication that there is a direct interaction between Eu(III) and the respective groups (–COO^−^ and –NH) that leads to the formation of inner-sphere complexes. Analogous observations were made previously for the adsorption of U(VI) on X-alginate beads [23].

**Figure 4:**
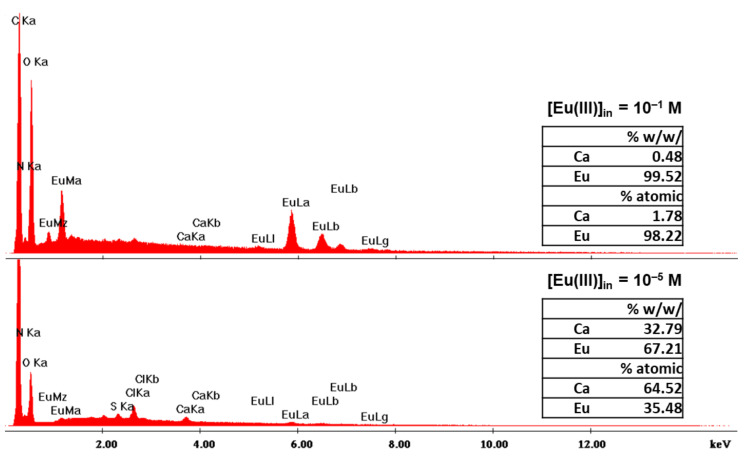
Energy-dispersive X-ray spectra (EDS) of dried X-alginate beads after sorption of different amounts of Eu(III) from solutions with different initial Eu(III) concentrations, as indicated.

An EDS analysis was performed on selected samples, i.e., on X-alginate beads that were used for adsorption from solutions with initial Eu(III) concentrations equal to 10^−5^ and 10^−1^ mol L^−1^ (Figure 4). The EDS analysis revealed that Ca is present in both samples, and that the atomic ratio Eu/Ca changes from 0.55 to 55, respectively. These results are consistent with a cation exchange process between Eu(III) and Ca(II). However, the moles of Eu(III) adsorbed are up to seven times more than the moles of Ca(II) present in the same mass of the X-alginate beads, which clearly shows that Ca(II)-coordination sites are not the only sites available for Eu(III) coordination; indeed, –COO−, –NH, and −OH groups on the framework of the aerogel can also act as coordination sites. Analogous observations were previously made for the adsorption of U(VI) on X-alginate beads [23].

**Figure 5:**
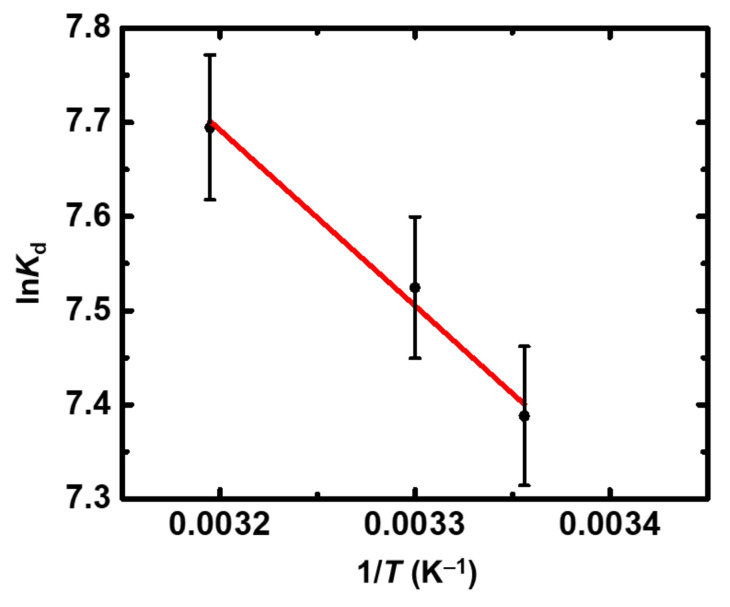
ln*K*_d_ as a function of 1/*T* for the sorption of Eu(III) by X-alginate aerogels at an initial Eu(III) concentration equal to 5 × 10^−4^ mol L^−1^. Experimental conditions are as follows: contact time 24 h, pH 3.0, adsorbent dosage 0.4 g L^−1^, agitation rate 125 rpm.

The data obtained from the temperature effect experiments are graphically presented in Figure 5. The calculated thermodynamic parameters (Δ*H*^o^ = 155.3 kJ mol^−1^; Δ*S*^o^= 113.7 J K^−1^ mol^−1^) clearly indicate that Eu(III) sorption is an endothermic and entropy-driven process, similar to the thermochemical behavior previously observed for U(VI) [23], suggesting a similar sorption mechanism based on inner-sphere complex formation between the surface moieties and Eu(III) cations.

### 2.2. Th(IV) Sorption

Thorium sorption experiments were also performed at pH 3 in order to obtain comparable results and to avoid extensive hydrolysis, polynucleation, and surface precipitation reactions. The concentrations used in this study ([Th(IV)] < 0.001 M) are below saturation but, at pH 3, Th(IV) exists in solution mainly in the form of the Th(OH)_2_^2+^ and Th(OH)_3_^+^ species [27]. The affinity of these Th(IV) species to form complexes is expected to be significantly reduced due to their significantly lower charge density compared to the non-hydrolyzed aquo cation (Th^4+^).

**Figure 6:**
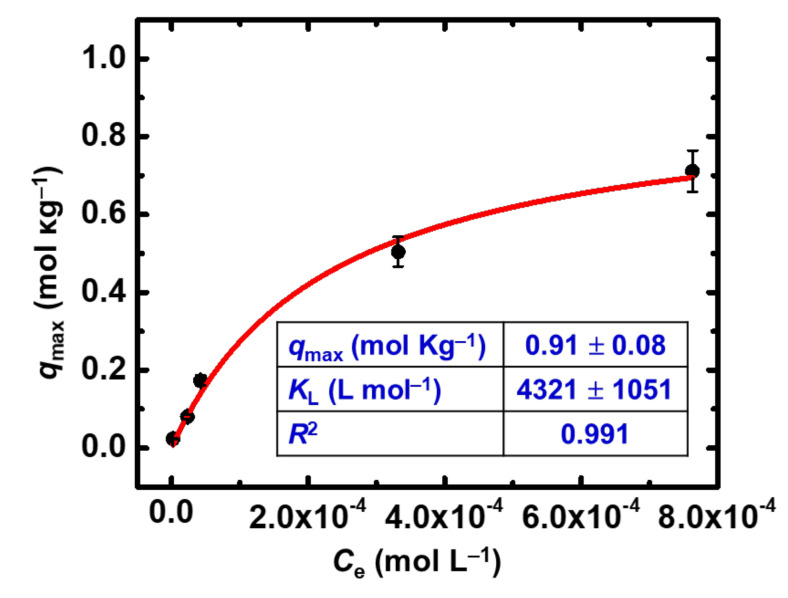
Experimental data and Langmuir sorption isotherm of Th(IV) on X-alginate aerogels at 283 K and varying initial Th(IV) concentrations (10^−5^–10^−3^ mol L^−1^). Experimental conditions are as follows: contact time 24 h, pH 3.0, adsorbent dosage 0.4 g L^−1^, agitation rate 125 rpm.

The maximum sorption capacity calculated using the Langmuir model (Figure 6) is *q*_max_ = 0.91 mol Th(IV) per kg of X-alginate aerogel (211 g kg^−1^). This value is significantly lower than the corresponding value for Eu(III) and U(VI) [23], in agreement with the fact that the effective charge plays a central role regarding the stability of the surface species. Nevertheless, this value is higher than the corresponding values obtained for modified biochars (20 g kg^−1^ < *q*_max_ < 176 g kg^−1^) [28,29,30,31,32,33]. Compared to other aerogel materials that have been reported in the literature for Th(IV) sorption (all graphene-based; Table 1) [34,35,36], X-alginate aerogels do not show the highest sorption capacity per weight but, interestingly, they show the highest sorption capacity per volume (27.9 g L^−1^; 6–12 times higher than the other aerogels). Thus, for example, the volume of X-alginate aerogel needed to adsorb a certain amount of Th(IV) is 6–12 times the volume of other aerogels.

**Figure 7:**
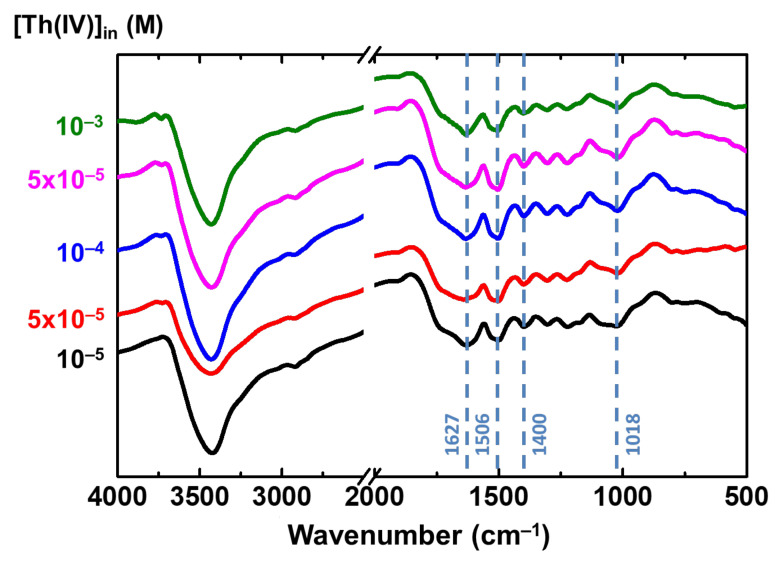
FTIR spectra of dried X-alginate beads after sorption of different amounts of Th(IV) from solutions with different initial Th(IV) concentrations (10^−5^–10^−3^ mol L^−1^, as indicated).

From the FTIR spectra shown in Figure 7, which correspond to dried X-alginate beads after sorption of different amounts of Th(IV), similar observations can be made to those described above for Eu(III), as in Section 2.1. The changes are not as intense as in the case of Eu(III) or U(VI), in agreement with smaller sorption capacity of Th(IV) compared to Eu(III) and U(VI), but they indicate again a direct interaction between Th(IV) species and the respective groups (–COO– and –NH) of X-alginate, resulting in the formation of inner-sphere complexes [23].

**Figure 8:**
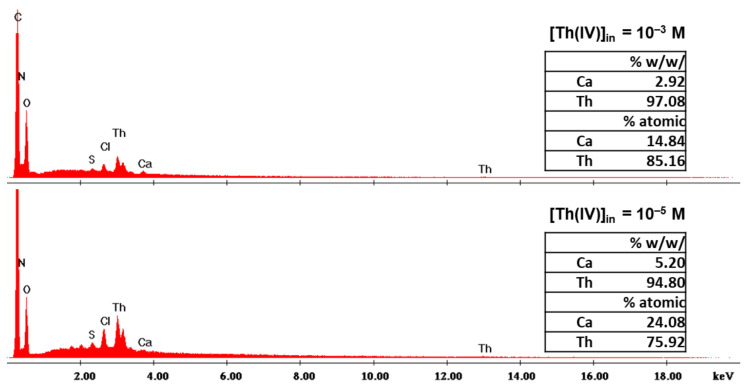
Energy-dispersive X-ray spectra (EDS) of dried X-alginate beads after sorption of different amounts of Th(IV) from solutions with different initial Th(IV) concentrations, as indicated.

An EDS analysis was performed on selected samples, i.e., on X-alginate beads that were used for adsorption from solutions with initial Th(IV) concentrations equal to 10^−5^ and 10^−3^ mol L^−1^ (Figure 8). The EDS results showed that the atomic ratio Th/Ca in the beads changes from 3.2 to 5.7, respectively. These results not only indicate cation exchange between Th(IV) species and Ca(II), but that Ca(II) are being replaced to a great extent even at low concentrations (i.e., 10^−5^ mol L^−1^), showing a great affinity of Th(IV) species to X-alginate. Again, Ca(II)-occupied sites are not the only coordination sites available for Th(IV) coordination, as the moles of Th(IV) adsorbed are up to 1.4 times more than the moles of Ca(II) present in the same mass of the aerogel. As has been previously discussed for Eu(III) (see Section 2.1) and U(VI) [23], –COO–, –NH, and −OH groups on the framework of the aerogel provide numerous sites available for coordination with Th(IV).

**Figure 9:**
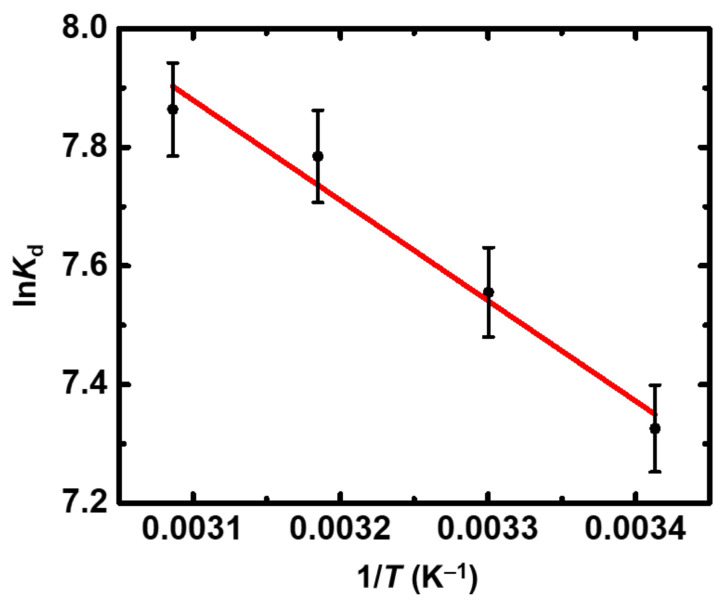
ln*K*_d_ as a function of 1/*T* for the sorption of Th(IV) by X-alginate aerogels at an initial Th(IV) concentration equal to 5 × 10^−4^ mol L^−1^. Experimental conditions are as follows: contact time 24 h, pH 3.0, adsorbent dosage 0.4 g L^−1^, agitation rate 125 rpm.

The thermodynamic parameters (Δ*H*^o^ = 140.6 kJ mol^−1^; Δ*S*^o^ = 109.2 J K^−1^ mol^−1^) obtained from the evaluation of the experimental data corresponding to the temperature effect (Figure 9) suggest that sorption is an endothermic and entropy-driven process. This is similar to the thermochemical behavior observed for U(VI) [23] and Eu(III) and indicates a similar sorption mechanism, which is based on the direct interaction between the surface moieties and the Th(IV) species.

### 2.3. Desorption Studies and Application to Wastewater Solutions

Eu(III) and Th(IV) desorption has been studied with batch-type recovery experiments, i.e., via extraction with an aqueous solution of EDTA (pH 10). The recovery was calculated using Equation (3), and it was evaluated to 65 ± 2% and 70 ± 3% for Eu(III) and Th(IV), respectively. After desorption, the beads remained apparently intact and could be reused for further sorption experiments.

Application of X-alginate aerogel beads to Th(IV) and Eu(III) recovery from wastewater solutions under similar conditions to de-ionized water test solutions have shown that, in contrast to Eu(III), which is only partially (~20%) adsorbed, Th(IV) is quantitatively (100%) removed from the solution. The latter can be attributed to the following facts: (a) Th(IV) under the given conditions (pH 8.1) exists in the form of neutral Th(OH)_4_ which presents very low solubility and high sorption tendency, and (b) the use of an extremely low Th(IV) concentration ([Th] = 0.1 nmol L^−1^) to avoid colloid formation and/or solid phase precipitation.

## 3. Conclusions

X-alginate aerogel beads derived from Ca-alginate gels and the aromatic triisocyanate Desmodur RE show high adsorption capacities for Eu(III) and Th(IV). The concentration range studied covers a wide range of Eu(III) and Th(IV) concentrations expected in acidic process waters and wastewaters. The experimental data were fitted to the Langmuir isotherm model and the maximum adsorption capacity, *q*_max_, was found to be equal to 3.6 mol (550 g) of Eu(III) per kg of aerogel and 0.9 mol (211 g) of Th(IV) per kg of aerogel. The lower sorption capacity for Th(IV) compared to Eu(III) is attributed to the net charge of the dominant species in solution under the given experimental conditions (pH = 3), which is Eu^3+^ for Eu(III), and Th(OH)_2_^2+^ and Th(OH)_3_^+^ for Th(IV). Evaluation of the thermodynamic data indicated that in both cases sorption is an endothermic, entropy-driven process. Furthermore, FTIR spectroscopy indicated the formation of inner-sphere complexes between the surface functional groups of X-alginate beads and Eu(III) or Th(IV) species, and EDS analysis confirmed the post-adsorption presence of europium and thorium in the adsorbent. The sorption of these *f*-metal species is very similar to the one observed previously for U(VI).

To the best of our knowledge, this is the first aerogel material used for the adsorption of Eu(III). Compared to other materials used for the sorption of Eu(III), which are mostly carbon-based, X-alginate aerogels show by far the highest sorption capacity. Regarding Th(IV) species, X-alginate aerogels show the highest capacity per volume (27.9 g L^−1^) among the aerogels reported in the literature. 

Both Eu(III) and Th(IV) could be recovered from the beads by 65% and 70%, respectively. Th(VI) could also be quantitatively removed from wastewater, while Eu(III) could be removed by 20%.

The above, along with their stability in aqueous environments, make X-alginate aerogels attractive candidates for water treatment and metal recovery applications. Future studies will focus on the application of X-alginate aerogels in packed columns and pilot scale water treatment units, which are of particular interest for the potential commercialization of the material.

## 4. Experimental Section

### 4.1. Materials and Methods

Sodium alginate PROTANAL LF 240 D (G/M = 0.43–0.54) was used as starting material. Then, Eu(NO_3_)_3_·6H_2_O (analytical grade), CaCl_2_ and Arsenazo-III were purchased from Sigma-Aldrich (Saint Louis, MO, USA), and Th(NO_3_)_4_·5H_2_O was purchased from Merck (Darmstadt, Germany). Desmodur RE (27% *w*/*w* triphenylmethane-4,4′,4″-triisocyanate (TIPM) solution in ethyl acetate) was generously provided by Covestro AG (Leverkusen, Germany). MeCN (HPLC grade) was purchased from Fisher Scientific (Waltham, MA, USA), and acetone (P.A., ISO reagent) was purchased from Lach-Ner (Neratovice, Czechia). All solvents were used as received.

All UV–Vis spectrophotometry measurements were carried out using a UV-2401 PC Shimadzu (Kyoto, Japan) spectrophotometer. Alpha-spectroscopy was performed using a high-resolution alpha-spectrometer (Alpha Analyst Integrated Alpha Spectrometer, Canberra, Meriden, CT, USA) equipped with semiconductor detectors. All pH measurements were performed using a commercial glass electrode (Sentek, Braintree, UK), which was calibrated prior to and after each experiment using a series of buffer solutions (pH 2, 4, 7 and 10, Scharlau, Barcelona, Spain). The FTIR spectroscopic measurements were carried out using an FTIR spectrometer 8900 (Shimadzu, Kyoto, Japan). Samples were prepared as pellets with KBr.

Energy-dispersive X-ray spectra (EDS) analysis was performed using an FEI Quanta Inspect (FEI Company, Hillsboro, OR, USA) scanning electron microscope (SEM) equipped with an EDAX EDS system.

### 4.2. Preparation and Characterization of X-Alginate Aerogels

X-alginate aerogels were prepared and characterized as previously described [14]. The concentration of the initial aqueous solution of sodium alginate was 3% *w*/*w*.

### 4.3. Adsorption Experiments

The adsorption experiments of Eu(III) and Th(IV) on X-alginate aerogels were conducted as described elsewhere [37,38,39]. Briefly, aqueous solutions (25 mL) containing 0.01 g (0.4 g L^−1^) of X-alginate aerogel beads and varying metal ion concentrations at pH 3.0 were prepared, and the adsorption of Eu(III) or Th(IV) (M(z)) was investigated under ambient conditions. The initial metal ion concentration was varied between 10^−5^ mol L^−1^ and 0.1 mol L^−1^ for Eu(III) and 10^−5^ mol L^−1^ and 0.001 mol L^−1^ for Th(IV). The contact time to reach equilibrium was set at 24 h and the determination of metal ions in solution was carried out using UV–Vis spectrophotometry directly at higher Eu(III) concentrations and using Arsenazo-III at lower Eu(III) and Th(IV) concentrations [40]. Thorium analysis was also performed by alpha-spectroscopy. The effect of temperature was investigated at 298 K, 303 K, 314 K, and 324 K at a metal ion concentration of 5 × 10^−4^ mol L^−1^. The spectrophotometric method was calibrated prior to and after each experiment using reference solutions, which were prepared by dissolution of analytical grade nitrate salts of the respective metal ions (i.e., Eu(NO_3_)_3_·5H_2_O) and Th(NO_3_)_4_·5H_2_O) in de-ionized water under similar conditions. The amount of M(z) adsorbed, *q*_e_ (mol kg^−1^), was calculated using Equation (2), where *C_o_* (mol L^−1^) is the initial M(z) concentration in the solution, *C_e_* (mol L^−1^) is the final M(z) concentration in the solution at equilibrium, *V* (L) is the solution volume, and *m* (g) is the weight of X-alginate aerogel beads. Equation (2) is as follows:

(2) $$q_{e}=\frac{\left(C_{o}-C_{e}\right)V}{m}$$

After adsorption, X-alginate beads were dried overnight in a vacuum oven at 80 °C and were characterized by FTIR (KBr) spectroscopy and EDS analysis.

The applicability of X-alginate aerogel beads to remove Eu(III) and Th(IV) from “real” samples was investigated by contacting wastewater obtained from a wastewater treatment plant in Nicosia contaminated with Eu(III) ([Eu] = 1 × 10^−4^ mol L^−1^) and Th(IV) ([Th-230] = 0.1 nmol L^−1^). Sampling was carried out according to the Standard Methods. Water sample analysis was performed using the Standard Methods for the examination of water and wastewater [41]. The uptake experiments were performed using 0.4 g of X-alginate aerogel beads per liter of solution.

### 4.4. Desorption Experiments

In this study, Eu(III) and Th(IV) batch-type recovery experiments were performed using an aqueous EDTA solution 0.1 M (pH 10). After the adsorption experiments, the beads were separated from the suspension and 30 mL of the EDTA solution were added. Subsequently, the new suspension was shaken for 7 h in a thermostatic orbital shaker (100 rpm, 23 ± 2 °C). Afterwards, the beads were separated and the europium or thorium (M(z)) concentration in solution was determined using UV–Vis spectrophotometry. The desorption efficiency (% Desorption) was determined using Equation (3) ([M(z)]_ads_ and [M(z)]_des_ are the concentrations of ions adsorbed and desorbed, respectively).

(3) $$\%\;Desorption=\frac{{\left[M\left(z\right)\right]}_{des}}{{\left[M\left(z\right)\right]}_{ads}}\times 100$$

## Figures and Tables

**Table 1:** Th(IV) sorption capacity from laboratory solutions and selected material properties of different aerogel adsorbents.

Material	*T* (K)	pH	Max. Sorption Capacity (Langmuir) *q*_max_ (mg g^−1^)	Langmuir Constant *K*_L_ (L mg^−1^)	BET Surf. Area *σ* (m^2^ g^−1^)	Bulk Density *ρ*_b_ (mg cm^−3^)	Max. Sorption Capacity *q*_max_ (mg cm^−3^)	Ref.
X-alginate aerogel	298	3	211.12	0.019	459 ^a^	150 ^a^	27.9	this work
graphene nanoribbons aerogel	298	3	380.4	0.020	597.4	6.2	2.36	[34]
poly(TRIM/VPA)-functionalized graphene oxide nanoribbons aerogel ^b^	298	3	457.9	0.045	433.2	10.6	4.85	[35]
PEI-functionalized graphene aerogel ^c^	298	2	38.17	0.0088	36.06			[36]

^a^ Data taken from ref. [22]. ^b^ Abbreviations are defined as follows: TRIM, trimethylolpropane trimethacrylate; VPA, vinylphosphonic acid. ^c^ The abbreviation PEI is defined as polyethylenimine.

## Data Availability

The data presented in this study are available on request from the corresponding authors.
